# Comparison of the effects of high-flow nasal cannula and bilevel positive airway pressure treatments as respiratory physiotherapy interventions for children with asthma exacerbation: a randomized clinical trial

**DOI:** 10.31744/einstein_journal/2024AO0588

**Published:** 2024-08-12

**Authors:** Maisi Muniz Cabral David, Evelim Leal de Freitas Dantas Gomes, Carla Lima Feitoza Cavassini, Josiane Germano Luiz, Dirceu Costa

**Affiliations:** 1 Post graduation program Science Rehabilitation Universidade Nove de Julho São Paulo SP Brazil Post graduation program Science Rehabilitation, Universidade Nove de Julho, São Paulo, SP, Brazil.; 2 Post graduation program Science Rehabilitation Universidade de São Paulo São Paulo SP Brazil Post graduation program Science Rehabilitation, Universidade de São Paulo, São Paulo, SP, Brazil.

**Keywords:** Asthma, Cannula, Physical therapy modalities, Length of stay, Bronchodilator agents, Positive-pressure respiration, Child, hospitalized, Child

## Abstract

**Objective:**

To evaluate and compare the efficacy of high-flow nasal cannula treatment and that of bilevel positive airway pressure treatment as respiratory physiotherapy interventions for pediatric patients who are hospitalized because of asthma exacerbation.

**Methods:**

During a randomized clinical trial, treatment was performed using a high-flow nasal cannula and bilevel positive airway pressure for hospitalized children with asthma. After randomization, data regarding lung function, vital signs, and severity scores (pulmonary index, pediatric asthma severity, and pediatric asthma scores) were collected.

**Results:**

Fifty patients were included in this study (25 in the Bilevel Group and 25 in the high-flow nasal cannula group). After 45 minutes of therapy, an improvement in the forced expiratory volume in 1 second was observed. The high-flow nasal cannula group required fewer days of oxygen (O_2_) use, used fewer bronchodilators (number of salbutamol puffs), and required shorter hospitalization periods than the Bilevel Group (6.1±1.9 *versus* 4.3±1.3 days; 95% confidence interval, -5.0 to -0.6)

**Conclusion:**

A high-flow nasal cannula is a viable option for the treatment of asthma exacerbation because it can reduce the hospitalization period and the need for O_2_ and bronchodilators. Additionally, it is a safe and comfortable treatment modality that is as effective as bilevel positive airway pressure.

**ClinicalTrials.gov Identifier:** NCT04033666.

## In Brief

A high-flow nasal cannula is a practical and safe instrument that can be used for children with asthma exacerbation and promotes beneficial outcomes such as improved asthma severity scores and reduced hospitalization durations, salbutamol use, and oxygen use.

## Highlights

■ Positive airway pressure may improve the severity of asthma exacerbation.

■ A high-flow nasal cannula can improve respiratory physiotherapy outcomes.

■ Positive airway pressure is indicated for moderate asthma exacerbation.

■ Bilevel positive airway pressure and high-flow nasal cannula use may improve the pulmonary function.

## Graphical abstract

**Figure:**
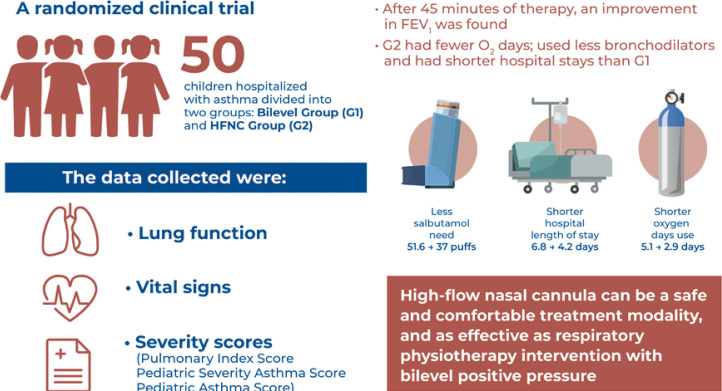

## INTRODUCTION

Asthma is a chronic, persistent, inflammatory respiratory disease^(1,2)^ that affects approximately 300 million individuals worldwide. Studies have reported that 25% of children and adolescents treated at large urban centers exhibit symptoms of asthma, which is one of the main causes of hospitalization during childhood, absenteeism from school, and physical inactivity.^(1,3)^

Asthma is characterized by airflow obstruction caused by bronchospasms of the smooth muscles of the bronchi and bronchioles and mucus accumulation.^(2)^ The clinical manifestations include recurrent cough, shortness of breath, wheezing, and tightness of the chest that is associated with obstructed airflow, which is partially reversible. Medicinal treatments include inhaled corticosteroids and bronchodilators.^(1)^

During periods of exacerbation, bilevel positive airway pressure can be used as supportive therapy to help reverse bronchospasms. Furthermore, when used for acute asthma, bilevel positive airway pressure can reduce the respiratory effort and increase the bronchodilator effect of inhaled albuterol.^(4,5)^ The physiological effects of bilevel positive airway pressure include reduced respiratory effort, improved gas exchange, improved alveolar ventilation, restoration of the functional residual capacity, improved minute ventilation, elimination of carbon dioxide (CO_2_), and prevention of orotracheal intubation.^(6-8)^ Additionally, positive airway pressure is considered an effective nonpharmacological tool for the treatment of asthma.

Bilevel positive airway pressure is widely used as asthma treatment; however, its level of evidence remains limited.^(1,9)^ Furthermore, it is associated with discomfort and intolerance related to the mask interface. The 2017 consensus of the European Respiratory Society (ERS)/American Thoracic Society (ATS) expressed uncertainty regarding the recommendation of positive airway pressure as noninvasive ventilation (NIV) for acute asthma. Although studies have recommended positive airway pressure as physiological support,^(10)^ and especially as a respiratory physiotherapy intervention, few clinical trials of pediatric patients have been conducted.^(4)^ Therefore, the use of a high-flow nasal cannula (HFNC) has gained attention in pediatric intensive care units.

During high-flow oxygen therapy with an HFNC, oxygen is heated, humidified, and delivered to the upper airway at a high flow rate (>2L/min), thus generating positive airway pressure with an adjusted fraction of inspired oxygen (FiO_2_).^(11-13)^

Two clinical trials have compared the use of an HFNC with conventional oxygen therapy. The study by Raeisi et al.^(14)^ found a more pronounced improvement in the HFNC Group than in the conventional oxygen therapy group. However, the study by Gauto Benítez et al.^(15)^ observed little difference between these treatment modalities. Furthermore, a retrospective cohort study of NIV and HFNCs found that the initiation of support with NIV was delayed with HFNCs, thus prolonging the hospitalization period.^(16)^

The tolerance of children may be greater with HFNC use. Furthermore, HFNCs can be used for pediatric populations during periods of exacerbation. However, there is still a gap in the literature regarding the degree of exacerbation that requires the initiation of HFNC use and the resulting improvements in different severity scores of children. One study evaluated different flow rates using HFNCs for infants with bronchiolitis and suggested that the mechanisms related to the decreased dead space in the upper airway could result in better CO_2_ elimination and increased pharyngeal pressure, which could be beneficial for patients. These physiological improvements are likely to decrease the efforts of patients and improve oxygenation. Furthermore, decreased respiratory distress scores and increased oxygenation were observed.^(17)^

Therefore, this study aimed to evaluate and compare the effectiveness of HFNC treatment and that of bilevel positive airway pressure treatment as respiratory physiotherapy interventions for children and adolescents who are hospitalized because of asthma exacerbation. The exacerbation severity scores and whether these treatments had any impact on oxygen use, bronchodilator use, and the length of hospitalization were assessed.

## OBJECTIVE

To evaluate and compare the efficacy of high-flow nasal cannula use and bilevel positive airway pressure as respiratory physiotherapy interventions for patients who are hospitalized because of asthma exacerbation.

## METHODS

This parallel, randomized, blind clinical trial of the treatment of exacerbated asthma using either bilevel positive airway pressure or the HFNC as the respiratory physiotherapy intervention was conducted at the Pediatric Hospital in São Paulo, Brazil. This study was conducted in accordance with the norms governing research involving human subjects stipulated in Resolution 466/2012 of the Brazilian National Board of Health, and it was approved by the *Universidade Nove de Julho*, São Paulo Ethics Committee (CAAE: 83135718100005511; #3224784).

Patients eligible for this study were admitted to the urgent care ward of the hospital after presenting to the emergency department. This study received structural, logistic, and orientation support from the Respiratory Functional Evaluation Laboratory of the *Universidade Nove de Julho* and *Universidade de São Paulo*.

All technical logistics equipment and specific materials were provided by Fisher & Paykel Healthcare. Sixty-seven children and adolescents who were eligible for enrollment in this study did not require hospitalization after receiving urgent care.

Patients were included if they had a confirmed diagnosis of asthma, were between 5 and 16 years of age, experienced bronchospasms, agreed to participate, signed an informed consent form, and provided a statement of informed consent signed by a legal guardian.

Patients were excluded if they were unable to understand or undergo any of the tests because of mental or physical limitations, experienced inflammatory, congenital, or ischemic heart disease, experienced intolerance to any of the administered treatments, and experienced severe respiratory failure that required ventilatory support (invasive or noninvasive).

### Interventions

The Bilevel Group received bilevel positive airway pressure comprising diaphragm re-education exercises (3 × 10 breaths) and 12cm H_2_O inspiratory positive airway pressure (IPAP) and 8cm H_2_O expiratory positive airway pressure (EPAP) delivered for 45 minutes, depending on the tolerance of the patient, using a face mask. IPAP was adjusted over time to acquire a tidal volume more than 5L/min and a better respiratory pattern.

The HFNC Group received treatment comprising HFNC use and a dose calculated based on the weight of the child (2L/kg/min for the first 10kg and an additional 0.5L/kg/min for each additional 1kg). Diaphragm re-education exercises (3 × 10 breaths) were followed by continuous HFNC use.

For both groups, the daily treatment sessions lasted 45 minutes throughout the hospitalization period. Vital signs, peak expiratory flow (PEF), FEV_1_, and severity scores collected before and after each session were assessed. On the last day of hospitalization, the assessment performed before discharge included all variables of the initial assessment.

### Outcomes

**Figure 1:**
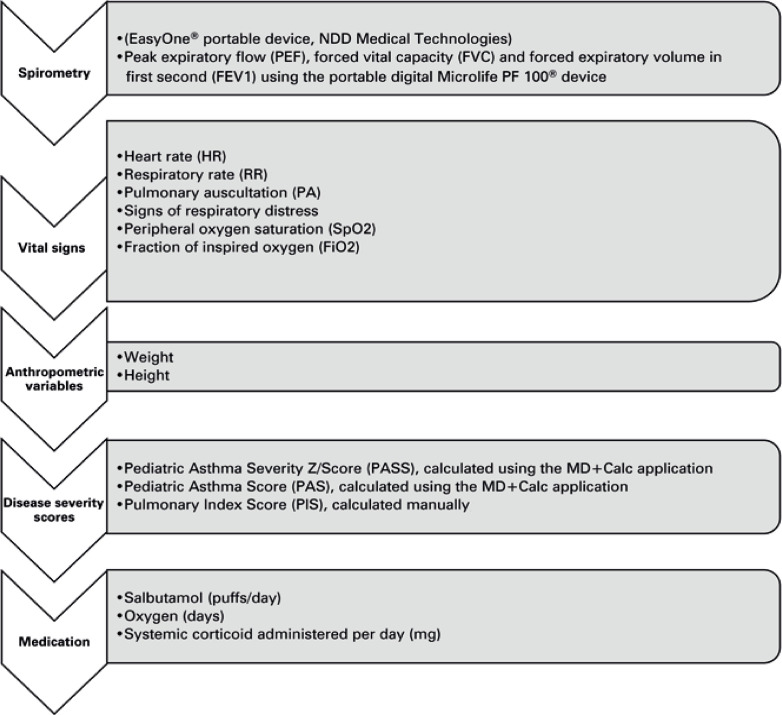
Evaluation sequence NIV: noninvasive ventilation; HFNC: high-flow nasal cannula.

The evaluations were performed three times, before treatment, after 45 minutes of treatment, and at the time of discharge from the hospital. The evaluations were conducted as shown in figure 1.

Examiner 1 collected data regarding vital signs, heart rate (HR), respiratory rate (RR), and peripheral oxygen saturation (SpO_2_). Additionally, examiner 1 performed pulmonary auscultation to calculate the following severity scores: pulmonary index score (PIS), pediatric asthma score (PAS), and pediatric asthma severity score (PASS).

The PIS is a clinical score based on observations of clinical signs such as the RR, wheezing, inspiratory-to-expiratory ratio, and signs of respiratory distress during the use of the accessory musculature. A score between 0 and 3 is assigned to each clinical sign, and the maximum score is 12. Seizure exacerbation was classified as mild (<7), moderate (7–11), or severe (>11).^(18)^

The PAS is based on the RR, SpO_2_, pulmonary auscultation, signs of respiratory distress (retractions), and dyspnea. Scores between 5 and 7 indicate mild exacerbation, those between 8 and 11 indicate moderate exacerbation, and those between 12 and 15 indicate severe exacerbation.^(19)^

The PASS is based on three clinical findings, wheezing, work of breathing (WOB), and the inspiratory-to-expiratory ratio. Each item is assigned a score of 1 or 2, and the maximum score is 6 points; however, if the score is more than 2, then the patient requires hospitalization.^(20)^

A spirometric analysis was performed to determine FEV_1_ (three acceptable readings depending on the patient’s tolerance) and PEF (three acceptable readings depending on the patient’s tolerance). All evaluations were performed while patients were in the seated position to preserve the clinical condition. Data regarding salbutamol (puffs/day), systemic corticosteroids (mg), and oxygen administered were also collected from the patients’ medical records.

Examiner 2 initiated 45 minutes of therapy plus respiratory exercises for each group. Examiner 1 evaluated the FEV_1_, PEF, PASS, PAS, PIS, pulmonary auscultation, HR, RR, SpO_2_, respiratory distress syndrome, and supplementary oxygen use of patients a second time. Evaluations were performed on the first day of hospitalization and during the morning of all subsequent days of hospitalization until discharge. We sought to maintain the comfort and well-being of the patients and respect the hospital routine. Evaluations of the HFNC Group were performed without treatment interruptions.

### Sample size

The sample size was calculated based on a pilot study of the following three main outcomes (all with 80% power and alpha of 0.05): PAS (difference of 2 points; standard deviation, 1.0 point); hospitalization period (difference of 2 days; standard deviation, 2.3 days); and puffs of salbutamol (difference of 11 puffs; standard deviation, 13 puffs). The minimum sample sizes were 16 per group for the PAS, 21 per group for the hospitalization period, and 22 per group for salbutamol puffs. To achieve the necessary number for all three outcomes and compensate for possible dropouts, the sample comprised 25 children and adolescents per group.

### Randomization

After both the patients and the legal guardians signed the consent forms, hospitalized children and adolescents eligible for the protocol were randomized into two groups using the randomization.com website. After block randomization, the interventions for the patients, either bilevel positive airway pressure (Bilevel Group) or HFNC treatment (HFNC Group), were written on papers that were placed in opaque envelopes. Evaluations were performed by a single examiner (examiner 1). Then, the respective interventions were performed by a single therapist (examiner 2). The patients were blinded to the other treatment group. The researcher who analyzed the data used a coded worksheet without group identifications so that the treatment groups were blinded.

### Statistical analysis

After verifying the normality of the data using the Shapiro-Wilk test, an unpaired Student’s *t* test was used for comparisons between groups, and the paired *t* test was used for the intragroup analysis.

To analyze outcomes with more than one occurrence, an analysis of variance followed by Tukey’s post hoc test were used for FEV_1_. An intragroup analysis of the severity score was performed using the Kruskal-Wallis test. Data were analyzed using Minitab statistical software 14 and expressed as the mean and standard deviation. The level of significance for the acceptance of statistical probably was set at 5% (p≤0.05).

The effect size was calculated using Cohen’s d, and the results were interpreted as follows;^(21)^ 0.21 to 0.49 indicated a small effect; 0.50 to 0.79 indicated a medium effect; and ≥0.80 indicated a large effect. An intention-to-treat analysis was performed for one patient who was randomized to the HFNC Group but required bilevel positive airway pressure. The results of HFNC and bilevel positive airway pressure treatments performed for this patient were not different.

## RESULTS

**Figure 2:**
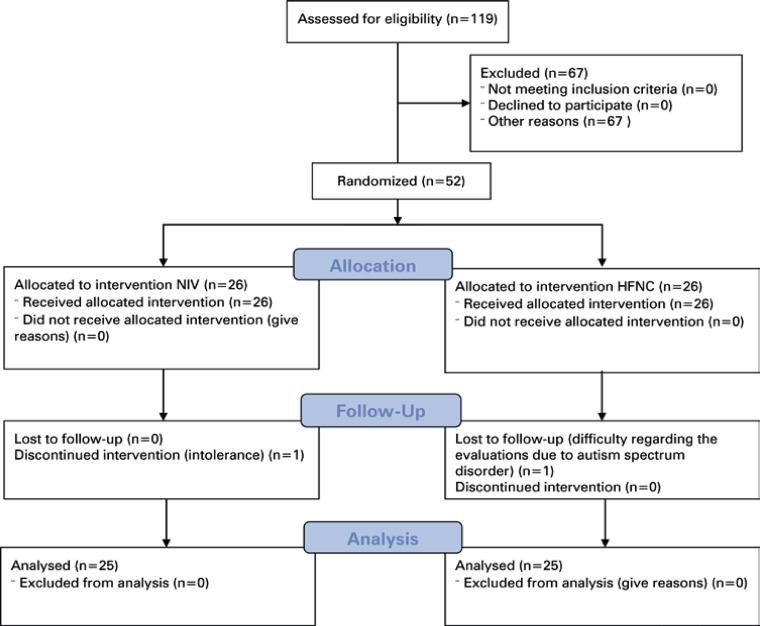
Patient randomization

Data of 52 patients with a diagnosis of bronchospasms who were hospitalized in the urgent care ward after presentation to the emergency department were collected (Figure 2). One patient in the Bilevel Group dropped out of the study because of intolerance to bilevel positive airway pressure; orotracheal intubation was required. None of the patients in HFNC Group required orotracheal intubation. Another patient who dropped out of the study experienced intolerance and difficulty during the evaluations.

The final sample comprised 50 patients (25 in the Bilevel Group and 25 in the HFNC Group). Table 1 present the general characteristics of the sample. These two groups had similar general characteristics. According to the severity scores, most patients had experienced an asthma attack classified as moderate at the time of hospitalization.

**Table 1:** General characteristics of the sample

	Bilevel Group (n=25)	95%CI Bilevel Group	HFNC Group (n=25)	95%CI HFNC Group	Adjusted mean difference of the 95% CI
Age (years)	7.2±1.9	(7.21–7.31)	7.62±1.8	(7.60–7.64)	(-1.09; 1.16)
Sex (M/F)	15/10	-	13/12	-	-
Height (cm)	129.7±10.1	(129.46–130.28)	126.7±10.7	(126.35–127.14)	(-9.05; 2.59)
Weight (kg)	29.4±7.2	(29.18–29.37)	28.4±8.2	(28.31–28.48)	(-5.72; 2.82)
Asthma attacks (within 12 months)	2.75±1.9	(2.74–2.75)	2.0±1.5	(2.04–2.05)	(-1.65; 0.19)
Hospitalizations (within 12 months)	1.25±1.05	(1.24–1.25)	1.4±1	(1.39–1.4)	(-0.61; 0.49)
Systemic corticosteroids (mg/6h)	24.85±6.75	(24.77–24.93)	25.84±7.26	(25.76–25.72)	(-3.64; 3.25)
FVC (%)	40±19.7	(39.87–40.12)	38.6±17.1	(38.49–38.73)	(-9.83; 12.57)
FEV_1_ (%)	32.11±12.1	(32.01–32.21)	31±١١.٣	(30.9–31.09)	(-6.83; 7.26)
FEF_25%-75%_	24.58±15.0	(24.51–24.66)	24.3±15.6	(24.25–24.40)	(-13.06; 5.38)
PEF (%)	34.41±19.3	(34.30–34.51)	31.9±17.6	(3.84–31.93)	(-12.35; 10.04)
PASS	3.19±0.67	(3.18–3.20)	3.68±1.05	(3.67-3.69)	(-0.11; 0.84)
PAS	10.47±1.4	(10.44–10.5)	10.78±1.65	(10.75-10.82)	(-0.65; 0.93)
PIS	5.42±1.07	(5.41–5.44)	5.94±1.74	(5.92-5.96)	(-0.48; 0.93)
Days of hospitalization	6.87±4.6	(6.85–6.89)	4.25±1.37*	(4.23–4.26)	(-5.00; -0.69)
Days of oxygen use	5.16±3.4	(5.15–5.18)	2.95±1.27*	(2.94–2.95)	(-3.52; -0.54)

*p<0.05.

95%CI: 95% confidence interval; F: female; FEF: forced expiratory flow; FEV1: forced expiratory volume in 1 second; FVC: forced vital capacity; HFNC: high-flow nasal cannula; M: male; PAS: pediatric asthma score; PASS: pediatric asthma severity score; PEF: peak expiratory flow; PIS: pulmonary index score.

The use of supplementary oxygen by both groups was compared in terms of the number of days of use during the hospitalization period. The HFNC Group used oxygen during a significantly fewer number of days (p=0.002); therefore, the HFNC Group required less oxygen than the Bilevel Group.

Bronchodilator use was measured based on the number of salbutamol puffs per day. The Bilevel Group used more salbutamol than the HFNC Group. The intragroup analyses indicated reductions in the use of salbutamol by both groups; however, the reduction was significantly greater in the HFNC Group (p<0.001).

The mean number of days of hospitalization attributable to asthma exacerbation was 4.35±1.37 days for the HFNC Group; however, it was 6.10±1.97 days for the Bilevel Group (Table 2).

**Table 2:** Data of the Bilevel and HFNC Groups

	Bilevel Group 1 (n=25)			HFNC Group (n=25)			Intragroup 95%CI	Intergroup 95%CI
	Before treatment	After 45 minutes of treatment	Discharge	Before treatment	After 45 minutes of treatment	Discharge		
FEV_1_ (%)	32.11±12.1 (32.01-32.21)	44.7±11.9^†^ (44-44.6)	66.6±30.3* (66.1-67)	31±11.3 (30.9-31.09)	38.5±9.6^†^ (38.4-38.9)	85.1±24.1*^†^ (85-85.9)	(-0.49; -0.23)	(1.17; 38.20)
FVC (%)	40±19.7 (39.87-40.12)	-	38.1±29.1 (38-38.7)	38.6±17.1 (38.49-38.73)	-	58.3±23.9*^†^ (57.9-58.5)	(-0.20; -0.01)	(6.30; 16.53)
FEF_25%-75%_	24.58±15.0 (24.51-24.66)	-	28±24.5	24.3±15.6 (24.25-24.40)	-	40.76±19.3*^†^	(1.03; 0.55)	(1.05; 25.49)
PEF (L/min)	78.8±32.8 (78.7-79)	90.9±31.2 (90.5-91)	114.8±22.2* (114.3-115)	76.5±30.2 (76.3-76.7)	98.4±24 (98.1-98.7)	126.8±39.9*^†^ (126.5-127.2)	(-115.60; -86.39)	(-32.92; -1.99)
PIS	5.42±1.07 (5.41-5.44)	4.38±0.6 (4.3-4.4)	0.25±0.4 (0.24-0.25)	5.94±1.74 (5.92-5.96)	4.68±1.18 (4.66-4.69)	0.6±0.7 (0.62-0.623)	(4.51; 5.91)	(-1.00;1.00)
PASS	3.19±0.67 (3.18-3.20)	2.53±0.5 (2.53-2.54)	0.08±0.2 (0.08-0.08)	3.68±1.05 (3.67-3.69)	2.88±0.7 (2.87-2.88)	0.19±0.4 (0.191-0.192)	(3.18; 4.01)	(-0.08; 0.33)
PAS	10.47±1.4 (10.44-10.5)	9.11±0.8 (9.08-9.14)	5.12±0.33 (5.1-5.14)	10.78±1.65 (10.75-10.82)	9.32±1.18 (9.29-9.34)	5.2±0.6 (5.27-5.3)	(4.69; 5.94)	(-1.00;1.00)
RR (breaths per min)	35.3±5.2 (35.3-35.7)	32.6±5.6 (32.4-32.9)	22.3±3.5* (21-23)	33.8±9 (33.7-33.9)	29.9±3.7 (29.8-30)	21±5* (18-22)	(10.12; 15.88)	(-2.46; 1.63)
HR (beats per min)	125.6±19.1 (125.3-126)	108.6±14.1 (108.4-108.7)	91.6±16.5* (90.9-91.9)	122.7±30.5 (122-123)	110.2±19 (109.8-110.5)	84.1±20.6* (84-86)	(24.50; 43.95)	(-4.21; 12.04)
SpO_2_ (%)	87.3±2.97 (87-88)	89.3±1.96* (89-91)	92±1.91* (92-94)	87.7±2.45 (87.5-88)	90±1.5 (89.7-90.2)	94.6±1.49* (94-95)	(-2.73; -1.51)	(-0.36; 1.75)
Systemic corticosteroids (mg/6h)	24.8±6.9 (24.7-24.9)	-	24.3±5.7 (24.3-24.4)	25.8±5.6 (25.7-25.9)	-	22.2±6.3* (22.2-22.3)	(1.85; 5.50)	(-6.59; 1.39)
Salbutamol (puffs)	61.04±16.2 (60.8-61.2)	-	51.6±11 (51.4-51.7)	55.5±20.4 (55.3-55.7)	-	37±8.9*^†^ (36.8-37)	(4.47; 20.9)	(8.8; 20.4)

*p<0.05 (45 min x discharge, two-way analysis of variance and Tukey’s post hoc test); † p<0.05 (unpaired intergroup analysis).

95%CI: 95% confidence interval; FEV_1_: forced expiratory volume in first second; FVC: forced vital capacity; HFNC: high-flow nasal cannula; HR: heart rate; PAS: pediatric asthma score; PASS: pediatric asthma severity score; PEF: peak expiratory flow; PIS: pulmonary index score; RR: respiratory rate.

**Figure 3:**
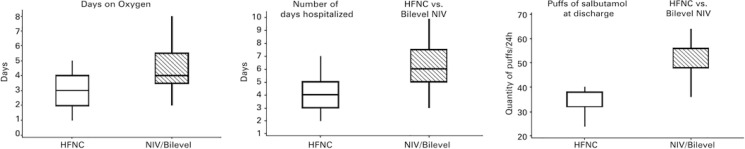
Days of oxygen use, days of hospitalization, and the average dose of bronchodilators at discharge HFNC: high-flow nasal cannula; NIV: noninvasive ventilation.

The bilevel group received an average of 2.73 hours (±1h) of therapy during hospitalization (45-minutes sessions). In contrast, the HFNC Group received an average of 90.5 hours (±35.5 hours) of therapy during hospitalization (p<0.001). This difference occurred because HFNC treatment was continuously administered and bilevel positive airway pressure treatment was administered only once daily (Figure 3).

## DISCUSSION

During the present study, HFNC treatment was administered as a respiratory physiotherapy intervention for acute asthma in children and adolescents. High-flow nasal cannula treatment was as effective as traditional bilevel positive airway pressure treatment. However, HFNC treatment was more advantageous because it reduced the need for bronchodilators (as measured by the number of puffs of salbutamol per day), the need for oxygen, and the hospitalization period. These findings demonstrate that HFNC respiratory physiotherapy can be as clinically advantageous as bilevel positive airway pressure for pediatric populations and indirectly lead to reduced hospital costs.

High-flow nasal cannula therapy has been applied more frequently during the past 10 years and has several clinical indications. High-flow nasal cannula therapy comprises a supply of a mixture of oxygen and humidified gas heated to approximately 37° with a flow rate more than 2L/min, thus causing an inspiratory flow of air to be delivered to the airway that is equal to or greater than the inspiratory flow of a spontaneously breathing patient.

Additionally, changes in respiratory mechanics, especially FEV_1_, after 45 minutes of treatment were observed. The bilevel group experienced greater improvement than the HFNC Group, and the forced vital capacity of the HFNC Group at discharge was significantly improved. In contrast, reductions in the HR and RR of both groups after 45 minutes of therapy were not significant. A difference in the FEV_1_ at 45 minutes was observed when bilevel positive airway pressure and HFNC were compared. The likely explanation was that the pressure levels established in the conductive airway through bilevel positive airway pressure were higher and the mask interface was able to pressurize the airway more effectively. The physiological explanation was the activation of the autonomic parasympathetic nonadrenergic and noncholinergic system; its association with mechanical bronchodilation has been explained in the literature.^(3,4)^

The improvement observed at discharge was significant, beyond what was expected, and greater in the HFNC Group than in the Bilevel Group. Regarding the severity scores, neither group exhibited improvements in the PIS, PAS, or PASS after 45 minutes of therapy. However, significant reductions were observed in both groups at discharge, thus demonstrating the clinical and therapeutic benefits of both techniques. The difference in the severity of the seizure classification by the PAS and PIS likely occurred because the PAS reflects a more detailed assessment of symptoms; therefore, we suggest using the PAS.

In 2018, Ballestero et al.^(22)^ found that HFNC therapy appears to be superior to conventional oxygen therapy for reducing respiratory distress within the first 2 hours of treatment for children with moderate-to-severe asthma exacerbation refractory to first-line treatment. However, the superiority of HFNC therapy over conventional oxygen therapy was not observed during two recent studies.^(14,15)^ Because HFNC treatment was administered continuously and bilevel positive airway pressure treatment was administered only once per day, there was a significant difference in the treatment duration.

Noninvasive treatments, such as continuous positive airway pressure and bilevel positive airway pressure, have been used more frequently for the treatment of pediatric acute respiratory distress during the past decade.^(23)^ Recently, HFNC has been added to this list of treatments;^(23-29)^ however, it had not been used previously for children and adolescents with asthma, and it had not been compared with bilevel positive airway pressure as a respiratory physiotherapy intervention.

The present results are in agreement with the findings described in the literature that indicate that bilevel positive airway pressure has the potential to decrease the rates of pneumonia and sinusitis, improve gas exchange, and result in some economic advantages compared to invasive mechanical ventilation. Bilevel positive airway pressure can reduce costs by reducing the hospitalization duration, thus minimizing possible additional interventions.^(2)^

Regardless of its modality, bilevel positive airway pressure leads to the relief of upper airway obstruction, promotes alveolar recruitment, improves the gas exchange, and consequently improves ventilation, perfusion, oxygenation, and the release of CO_2_. Studies have reported that HFNC treatment can optimize lung aeration and improve parameters such as oxygenation.^(17,30,31)^

Long before the emergence of HFNC treatment,^(31)^ bilevel positive airway pressure treatment was administered to children with asthma, and improvements in severity scores and WOB were observed in this population.^(30,32-34)^ Traditional bilevel positive airway pressure promoted a significant reduction in WOB, improved gas exchange, and diminished respiratory muscle effort in children with acute respiratory failure, indicating positive physiological and therapeutic effects. Despite these advantages of bilevel, and despite the fact that its nasal mask interface is well-tolerated because of its smaller dead space in comparison to that of other masks, leakage through the oral cavity is a considerable limitation of this method. Facial and oronasal masks result in less leakage but greater asynchrony, intolerance, and discomfort.^(34,35)^

Although HFNC treatment is a relatively new modality, its indications are increasing. Therefore, this technique is becoming more common as a conservative artificial ventilation method. Its indications for adults with a diagnosis of hypoxemic respiratory failure caused by pneumonia, those who have undergone extubation, those who received oxygenation prior to intubation, and those with acute pulmonary edema have been well-established in the literature. Furthermore, HFNCs have been used to treat bronchiolitis in infants;^(17)^ however, other indications, such as asthma in children, have also been studied^(36)^ (as in the present study).

The results of this study demonstrate that HFNC treatment is efficient, noninvasive, and has different mechanisms of action. High-flow nasal cannula can transmit positive airway pressure, optimize oxygenation and ventilation, and reduce WOB. Additionally, HFNCs can reduce the dead space of the nasopharynx with the depletion of oxygen and clearance of CO_2_, thereby ensuring a higher FiO_2_ compared to that achieved with conventional oxygen therapy modalities, thus enabling the flow of humidified, heated gas that generates greater pulmonary compliance, reduces airway resistance, and promotes the clearance of secretions.^(24,26,36)^ Therefore, we suggest that HFNC treatment is a safe and effective option for children and adolescents who are hospitalized because of mild-to-moderate asthma exacerbation.

High-flow nasal cannulas are expensive tools that have numerous benefits for hospitalized children and adolescents. When used for patients during asthma exacerbation classified as moderate, this noninvasive treatment modality can reduce the hospitalization duration, minimize complications, and enable more interactions and socialization between the patients and the therapist, health team, and family. Moreover, despite the high cost of treatment, HFNCs offer important savings by diminishing the use of inhaled corticosteroids and oxygen and shortening the hospitalization duration. Therefore, further studies of HFNC treatment should be performed.

Respiratory physiotherapy was administered in the hospital from the time of presentation to the emergency department until discharge. Resources are normally divided into instrumental and manual and used to treat dysfunctions related to obstructions and pulmonary restrictions. Obstructions are more common in the pediatric population. When these obstructions are not resolved promptly, they progress to restrictive conditions.

Patients with asthma first experience bronchospasms and edema, and mucus accumulates a few days later. Both bilevel positive airway pressure and HFNC treatments promote airway pressurization through different mechanisms, thus helping to resolve dysfunctions and optimize drug action. Based on these results, HFNC treatment as a resource for the hospital respiratory physiotherapy team provided considerable benefits.

### Study limitations

This study has limitations that prevented a more in-depth analysis of the results. Blood gas levels analyses were not performed because they are invasive and painful. FEV_1_ and PEF data were collected at the time of exacerbation; therefore, obtaining measurements was difficult. High-flow nasal cannula treatment was administered continuously, and bilevel positive airway pressure was administered once daily. Another limitation was the lack of severity scores, which have been validated in Portuguese, to assess asthma attacks. Although translations exist, the entire linguistic process has not been completed. A final limitation was the lack of a group who received only drug treatment. However, such a comparison has been performed among adult patients with asthma during a study that found that adding NIV to drug treatment resulted in superior outcomes similar to those observed during the present study.

## CONCLUSION

Based on these findings, high-flow nasal cannula therapy may be a viable and safe option for children and adolescents with asthma exacerbation. Furthermore, high-flow nasal cannula may assist with drug treatment when the patient cannot tolerate noninvasive ventilation. High-flow nasal cannula treatment appears to reduce the use of oxygen and bronchodilators; however, additional studies are necessary.
